# Electrocardiographic markers of myocardial infarction size, transmural extent, and extent of nonviable myocardium - comparison to CMR

**DOI:** 10.1186/1532-429X-18-S1-P78

**Published:** 2016-01-27

**Authors:** Daniel C Lee, Christine M Albert, Dhiraj Narula, Alan H Kadish, Andi Schaechter, Edwin Wu, Jeffrey J Goldberger

**Affiliations:** 1 grid.465264.7Northwestern University Feinberg School of Medicine, Chicago, IL USA; 2 grid.62560.370000000403788294Brigham and Women's Hospital, Boston, MA USA; 3 Quintiles Cardiac Safety Services, Mumbai, India; 4 Touro College and University System, New York, NY USA

## Background

Myocardial infarction **(MI)** size is an important determinant of mortality in post-MI patients, but the current gold standard test, cardiovascular magnetic resonance imaging **(CMR)**, is expensive and not widely available. We sought to determine whether information from a readily available standard 12-lead electrocardiogram **(ECG)** could be utilized to estimate infarct size, extent of transmural infarction, and extent of nonviable myocardium on CMR.

## Methods

Patients with a clinical history of MI enrolled in the DETERMINE and PREDETERMINE Trial and Registry (ClinicalTrials.gov ID NCT02164058, NCT01114269) were included. Patients with left bundle branch block were excluded. ECG's were analyzed for candidate ECGs markers, which may signify the presence and extent of MI [Q waves **(Qw)**, fragmented QRS **(fQRS)**, and T wave inversion **(TWI)**]. Contiguous Qw **(cQw MI)** and TWI **(cTWI)** required involvement of two leads in a coronary distribution. Qw, fQRS, and TWI in individual leads were also examined. CMR infarct mass as a percentage total left ventricular (**LV**) mass (**Infarct%**) and LV ejection fraction (**LVEF**) were planimetered from late gadolinium enhanced (**LGE)** and cine short axis stacks, respectively. LGE images were also scored visually on a 17-segment model for the total number of segments with MI that were transmural at any point (**Transmural Segments**) and the total number of segments that were >50% infarcted (**Nonviable Segments)**.

## Results

**Figure 1:**
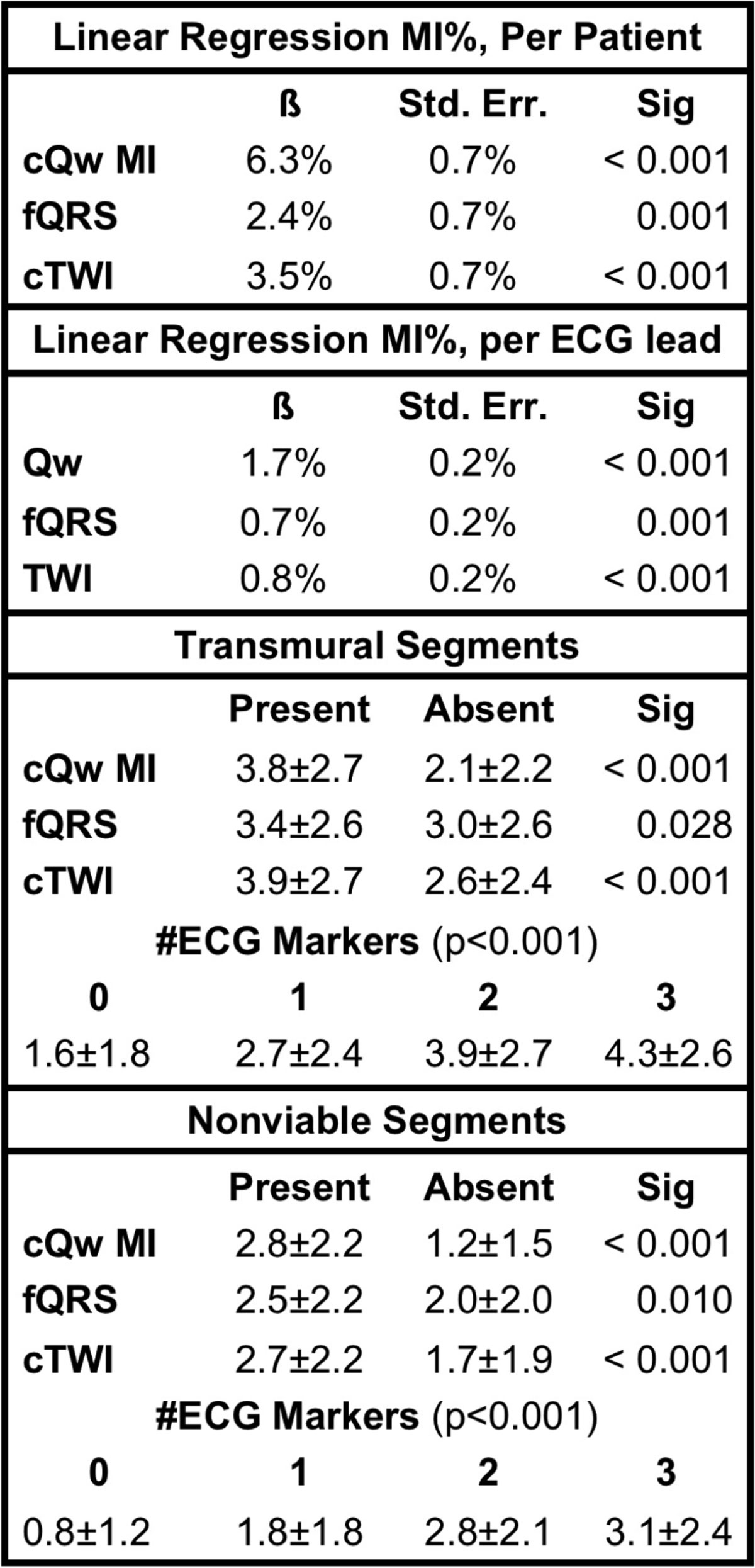
**Impact of ECG markers on MI size, transmural extent, and extent of nonviability**.

Of the 550 patients (mean age 61.5 ± 10.9 years, 78% men, mean LVEF=40 ± 11%) included in the analysis, 332(60%) had cQw MI, 216(39%) had fQRS, 235(43%) had cTWI, and 101(18%) had none of these ECG markers. Patients without any ECG markers had an infarct size of 9.0 ± 6.4% as compared to 14.0 ± 7.7%, 18.7 ± 9.2%, and 20.3 ± 9.7% for those with one (n = 179), two (n = 206), or all three (n= 64) ECG markers (p < 0.001). By multivariate linear regression, the presence of cQwMI, fQRS, and cTWI were independently associated with an increase in MI% by 6.3%, 2.4%, and 3.5%, (p≤0.001). On per lead analysis, multivariate linear regression demonstrated a significant continuous relationship between MI% and the number of leads affected by Qw, fQRS, and TWI. MI% was 7.8% when all leads were free of ECG markers and increased by 1.7%, 0.7%, and 0.8% for each lead affected by Qw, fQRS, and TWI (p≤0.001). The number of transmural segments and the number of nonviable segments was significantly higher in patients with cQW MI, fQRS, and cTWI than those without these ECG markers (see Figure 1). Also, transmural and nonviable segments increased with increasing number of ECG markers.

## Conclusions

Qw, fQRS, and TWI on ECG are independently associated with an increase in MI% measured by CMR in patients with a history of MI. Patients with these ECG markers also have a greater extent of transmural MI and nonviable myocardium, which increases with each additional marker seen on ECG. ECG estimates of MI size and extent may be useful to guide further risk stratification for sudden cardiac death.

